# Strength and fractal damage characteristics of cement-coal slag stabilized soil under freeze-thaw cycles

**DOI:** 10.1038/s41598-025-04449-8

**Published:** 2025-07-29

**Authors:** Kun Ren, Mengqi Shi, Mingshuang Liang

**Affiliations:** School of Transportation Engineering, Dalian Jiaotong University, Dalian, 116028 Liaoning China

**Keywords:** Cement-coal slag stabilized soil, Freeze-thaw cycles, Mesostructure, Fractal dimension, Strength, Damage, Engineering, Civil engineering

## Abstract

Triaxial compression tests and CT scanning experiments were conducted to study the variation law of the mechanical characteristics of cement-coal slag stabilized soil (CCSSS) and divide the meso-structure of the CCSSS into regions based on the distribution of CT values. The evolution law and fractal characteristics of the meso-structure in the damage zone under freeze-thaw cycles were analyzed, while the relationship between the macroscopic mechanical parameters of CCSSS and the morphology of the damage zone was established by fractal characteristics. The results showed that with an increase in the number of freeze-thaw cycles, the shear strength of CCSSS exhibited an exponential decay pattern. The freeze-thaw cycles significantly impacted the strain energy density and cohesion of CCSSS, while the change in internal friction angle is insignificant. As the number of freeze-thaw cycles increases, the damaged area of CCSSS gradually expands, with the effects of the initial 5 freeze-thaw cycles being more pronounced. Moreover, with an increase in water content, the damaged area of CCSSS showed an accelerating growth trend. There is a strong negative linear correlation between the fractal dimension of the damaged area and shear strength, accurately reflecting the damage caused by freeze-thaw action to the soil.

## Introduction

China has a widespread distribution of seasonal frozen soil, covering 53% of the national land area^1^.In the seasonally frozen regions, the subgrade of high-speed railways is susceptible to frost damage due to freeze-thaw cycles, leading to deteriorated mechanical properties of the fill materials, frost heaving deformation, and ultimately affecting the smooth operation of the high-speed railway^2^^3^. Conventional chemical stabilizers such as cement, lime, and fly ash were demonstrated to promote cementation of soil particles, thereby enhancing the mechanical characteristics^4–7^, especially cement showing superior enhancement. Despite its benefits, cement-stabilized soil exhibits inferior freeze-thaw stability, representing obvious deterioration in mechanical properties after multiple freeze-thaw cycles and significantly impacting the operational safety of the railway line^8,9^. To address this issue, research on enhancing the freeze-thaw stability of cement-stabilized soil through compositely adding new material garnered considerable interest from researchers both domestically and internationally.

Coal slag was demonstrated as a recent, potential, and economical fill material for chemically stabilized soil techniques, while it needs high utilization efficiency coupled with proper disposal practices to avoid significant environmental contamination^10,11^. Coal slag stabilized soil demonstrates significant improvements in mechanical properties, specifically demonstrated on expansive soils in numerous studies^12–15^. Coal slag fines impart a mechanical bonding within the soil, forming floccules and a porous nature^16^. These features improve soil mechanical performance while mitigating frost heave effects by providing pore space for water expansion. Additionally, coal slag enhances soil resistance to freeze-thaw cycles, with optimal coal slag content effectively reducing strength deterioration caused by freeze-thaw cycles^17^. Compared to conventional stabilizing agents, coal slag represents superior cost-effectiveness due to its origin as either natural or industrial waste residue^12^. Notably, combining coal slag with another stabilizer such as fly ash or cement at appropriate content shows superior effects of soil reinforcement^16^. However, as coal slag stabilized soil is a recent research field, current studies focus on expansive soil, lacking tests on other soil types. Furthermore, some areas such as long-term performance and the relationship between macroscopic mechanical parameters and mesoscopic damage in coal slag stabilization require deeper study.

The impact of freeze-thaw cycles on soil, as a primary factor leading to soil deterioration in seasonal frozen regions, has been extensively studied^18–20^. Shear strength and its associated parameters serve as crucial indicators for describing shear failure, and they are typically ascertained via triaxial tests. Research indicates that for subgrade soils in seasonal freezing regions, these parameters are influenced not only by soil particle composition and microstructure but also by water content and the frequency of freeze-thaw cycles^21^. Owing to variations in the studied soil types, added stabilizers, and designed experimental variables, different studies have revealed diverse patterns of changes in shear strength and its parameters. A portion of research^21–23^ reported an increase in the number of freeze-thaw cycles, and the shear strength, cohesion, and internal friction angle decreased. However, Fang et al.^24^ found the shear cohesion, and internal friction angle increased as the freeze-thaw cycles increased. Some studies found more intricate patterns of shear strength and its parameter change, Ding et al.^25^ reported the shear strength first increased and then decreased with the increase in freeze-thaw cycles. Tang et al.^26^ indicated that as the number of freeze-thaw cycles increases, the cohesion of the soil-rock mixture generally decreases first, then increases, and finally decreases, with the internal friction angle showing no apparent change. Liu et al.^27^ soil cohesion exhibited a decline with accumulated freeze-thaw cycles, while the internal friction angle displayed an initial decrease followed by an increase through freeze-thaw cycle tests on silt. It is noteworthy that some studies have suggested that as the number of freeze-thaw cycles increases, the impact of individual cycles gradually diminishes^28,29^. However, there is still limited research on the improvement effect of coal slag on the mechanical properties of subgrade soil, and further studies should be conducted.

The study of soil meso-structure can characterize the evolution of pores and the structural distribution in soil under freeze-thaw cycles. In terms of soil meso-structure, scanning electron microscope (SEM), nuclear magnetic resonance (NMR) technology, and computed tomography (CT) has been widely applied^30,31^. CT can continuously and non-destructively identify the meso-structural information inside the soil, observe the pore distribution and damage structure of the sample, and provide technical support for the visual characterization, detailed description, and quantitative analysis of the meso-damage structure inside the soil^32^. Zhao et al.^33^ using CT results demonstrated that the freeze-thaw cycle leads to an increase in porosity, which was believed to be the primary reason for the decrease in specimen strength. However, Zhang et al.^34^ through CT discovered that the porosity of the clay-gravel mixture sample repeatedly increased and decreased as the number of freeze-thaw cycles increased. Whereas the microscopic structural image lacks quantitative analysis, fractal dimension is a key parameter in fractal theory, and it has been proven to characterize pore evolution quantitatively and be correlated with damage evolution in soil^35^. Shu et al.^36^ used fractal dimensions to reflect the crack propagation and failure mechanism of saline-alkali soil. Shi et al.^37^ used the fractal dimension to ascertain the damage parameters of the sample, thereby aiding in the establishment of a constitutive model for the damage parameters of rubberized cement soil. Li et al.^38^ reported fractal dimension of size is strongly correlated with the hydration products in the solidified supersulfate saline soil. As the amount of curing agent increases, the hydration reaction becomes more pronounced, and the shape of the hydration products tends to become more complex.

Current research predominantly focused on unilateral investigations into either the mechanical property degradation of geotechnical materials under freeze-thaw cycles or their micro- and meso-structural analyses. However, establishing connections between macro and micro-scale parameters remains challenging. This study aims to investigate the meso-structure evolution and fractal characters of CCSSS under freeze-thaw cycles through triaxial consolidation undrained experiments and CT scanning experiments. Moreover using the fractal dimension of the damaged zone in CCSSS as a mediator, the relationship between macroscopic mechanical parameters of the CCSSS and the evolution of its meso-structure is established. The conclusions of this study can offer valuable reference for engineering construction in seasonal frozen regions.

## Experimental materials and methods

### Experimental materials and sample preparation

**Fig. 1:**
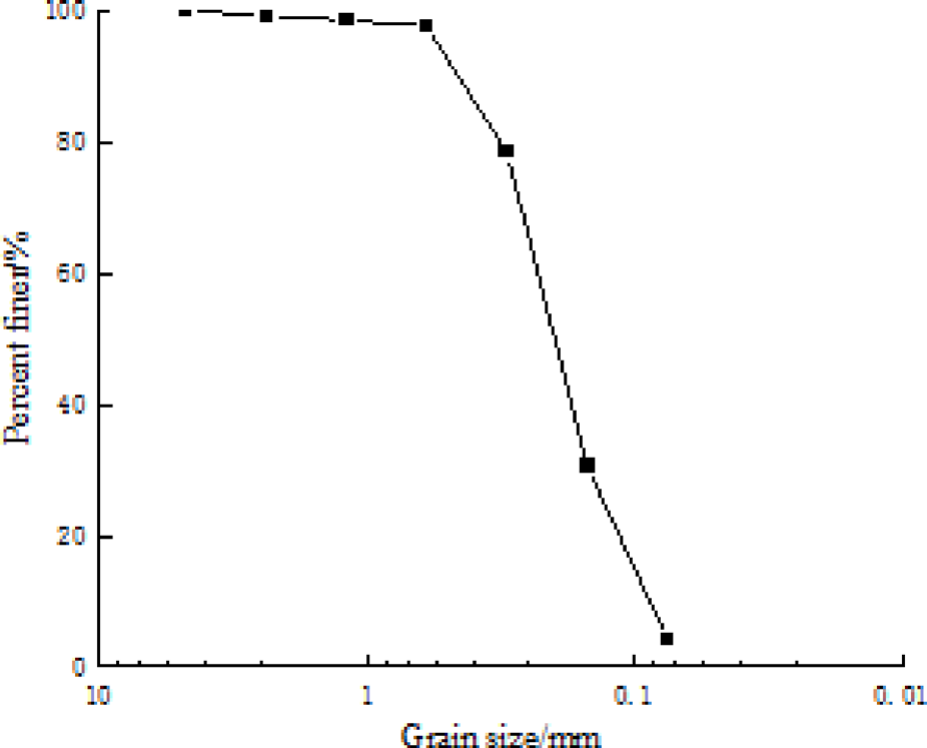
The grain gradation curve of the soil.

The aeolian sand soil provided for the study was sourced from the Fuxin to Zhangwu section of the Beijing-Shenyang Passenger Dedicated Line. It demonstrated a relatively concentrated particle size distribution, categorizing it as low-quality fill material, with its corresponding grain gradation curve plotted in (Fig. 1). The coal slag employed consists of waste slag from a coal-fired boiler in a thermal power plant, primarily comprising SiO_2_ and Al_2_O_3_ and the cement utilized is ordinary portland cement of grade 42.5.

**Fig. 2:**
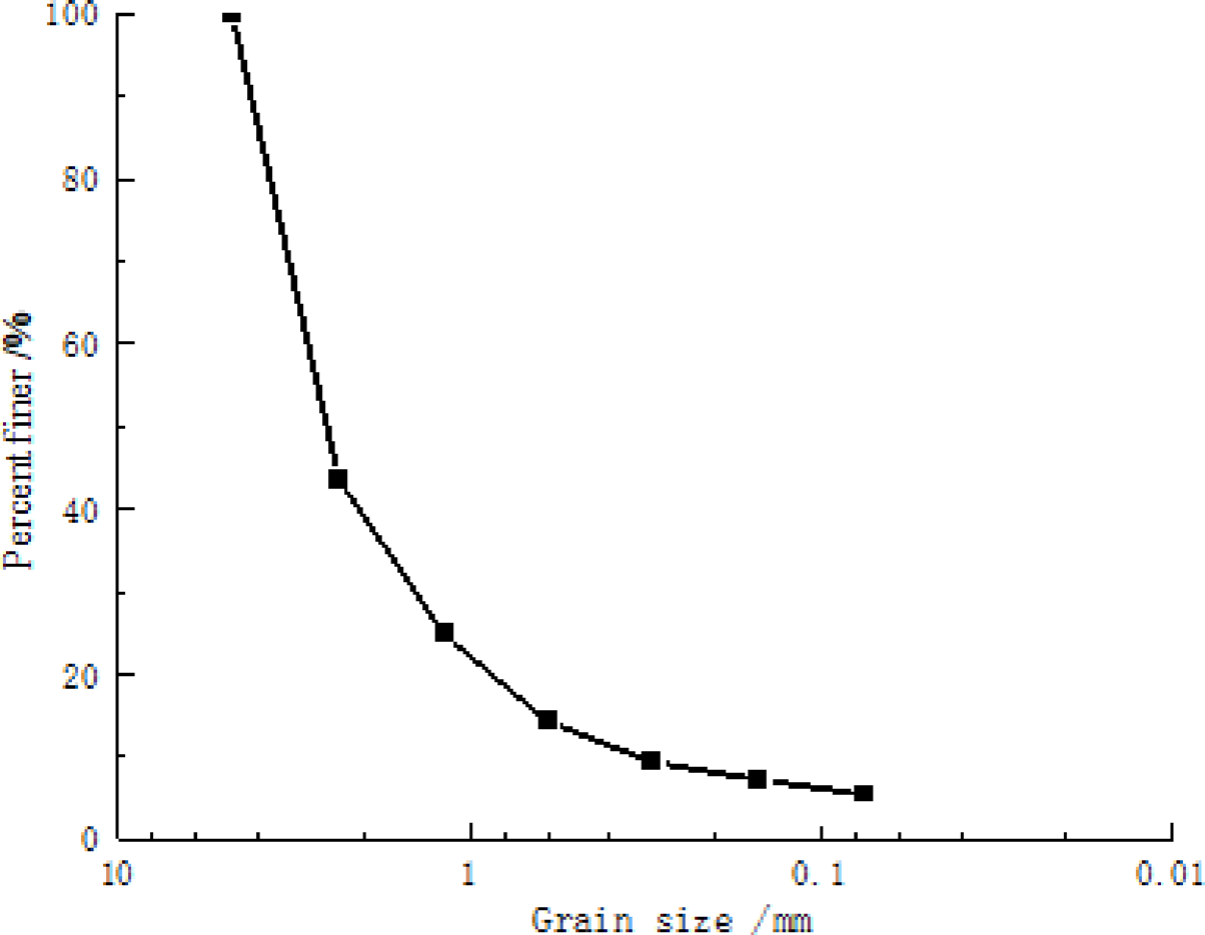
The grain gradation curve of the coal slag.

**Fig. 3:**
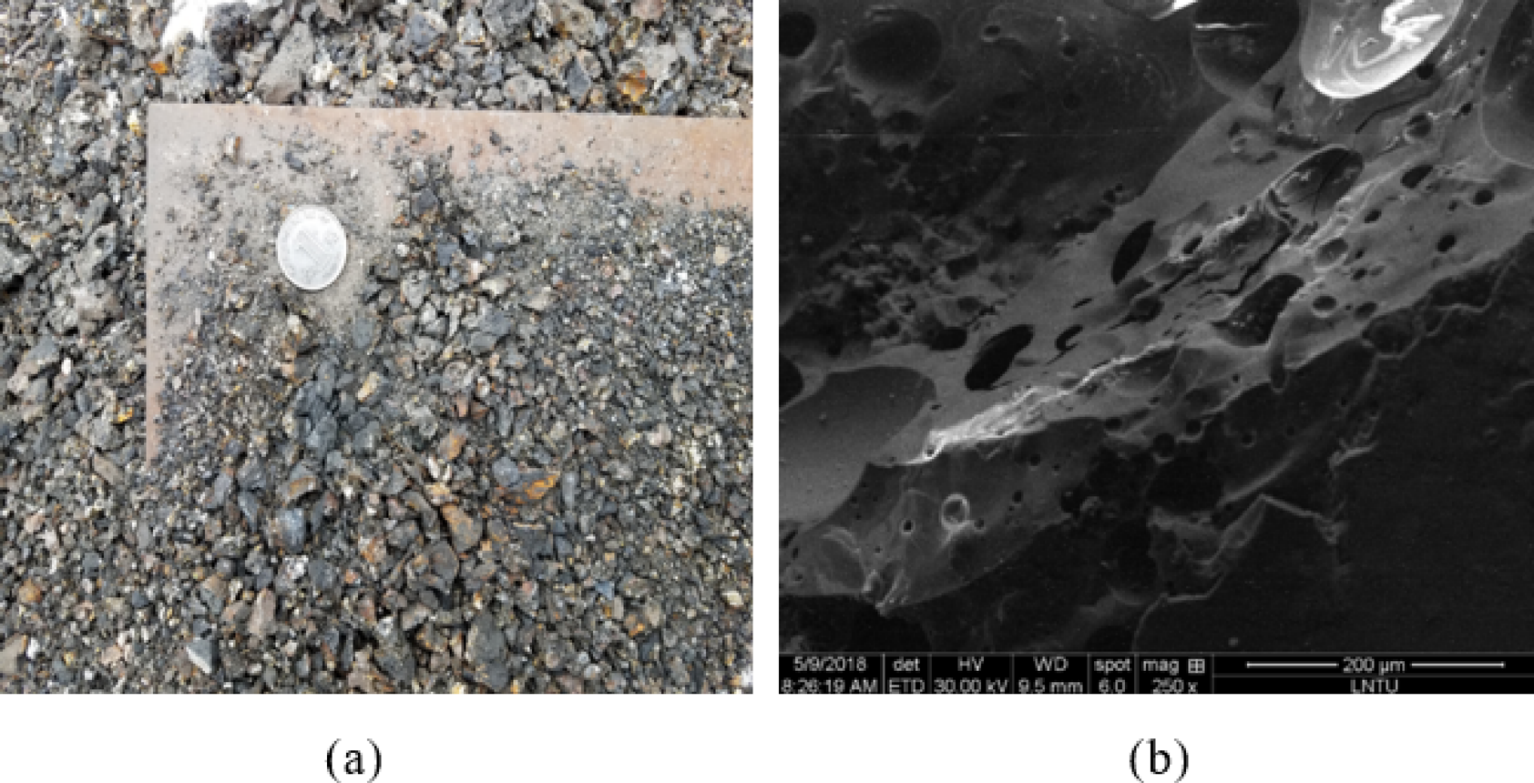
The structure of the coal slag. (**a**) crushing coal slag, (**b**) SEM image of coal slag.

The coal slag stabilizing the soil from the heating company in Fuxin City, as industrial pollution, was subjected to crushing, with its corresponding grain gradation curve plotted in (Fig. 2). The surface of coal slag is highly irregular, containing numerous open and closed pores, even being crushed into tiny particles. Figure 3 shows the macroscopic shape and micro-structure through SEM scanning of coal slag.

**Fig. 4:**
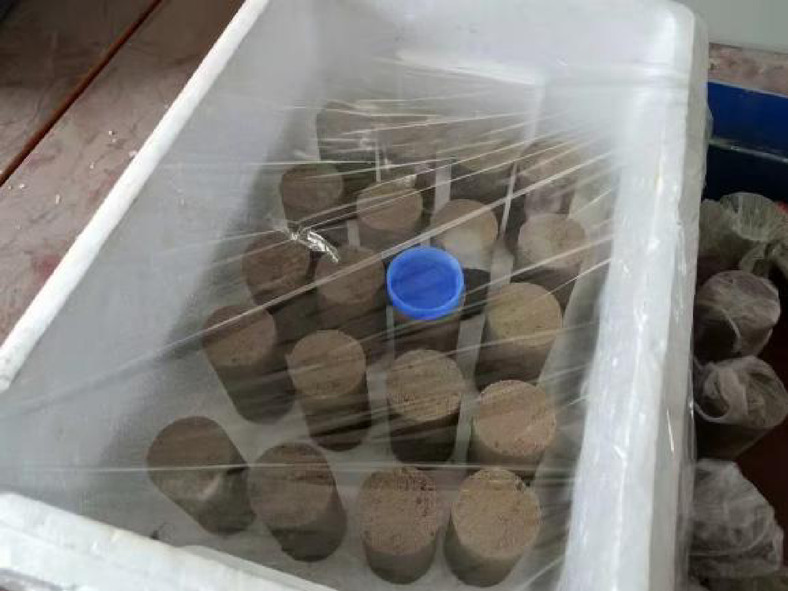
The process of triaxial samples preparation.

Excellent freeze-thaw stability has been demonstrated on coal slag, particularly at a 15% content of coal slag^39^. In this study, the mass ratio of cement, dried coal slag, and aeolian sand soil samples was 5:15:80. The optimum water content of 13.48% and the maximum dry density of 1.86 g/cm^− 3^ were determined through compaction tests. During the mixing process, to avoid water evaporation resulting in a water content below the designed value after curing, we increased the water mixing ratio. The resulting mixture was then layered into a tripartite saturator measuring Φ39.1 × 80 mm in 5 distinct layers, compacted through vibration and cured for 7 d under standard conditions (20 ± 2℃, humidity ≥ 95%). Figure 4 shows the process of triaxial samples preparation.

### Experimental methods

To investigate the impact of water content on the freeze-thaw resistance of CCSSS, samples with varying water contents were prepared after curing. Water was dripped onto the samples using a rubber dropper, and then the water content of each group was measured, yielding three groups with water contents of 11.48, 12.83, and 14.47%. To ensure uniform distribution of water, immediately cover it with plastic film after adding water and let it sit for 24 h. Among these, 12.83% closely approximates the optimal water content. Additionally, to investigate the freeze-thaw resistance of CCSSS when saturated, a group of samples underwent soaking treatment, resulting in a group with a water content of 16.02%. Based on local meteorological data, the freezing temperature was established at −15℃ with a duration of 12 h, while the thawing process occurred at room temperature of 20℃ over a period of 12 h. To mitigate moisture loss resulting from the freeze-thaw cycle, the modified soil sample was encased in plastic film prior to the experiment. The detailed experiment procedure for the freeze-thaw cycle is outlined in (Table 1).

**Table 1:** Freeze-thaw tests scheme.

No.	Water content W/%	Freeze-thaw cycles *N*
1	11.48	0, 1, 3, 5, 7, 10
2	12.83	0, 1, 3, 5, 7, 10
3	14.47	0, 1, 3, 5, 7, 10
4	16.02	0, 1, 3, 5, 7, 10

**Fig. 5:**
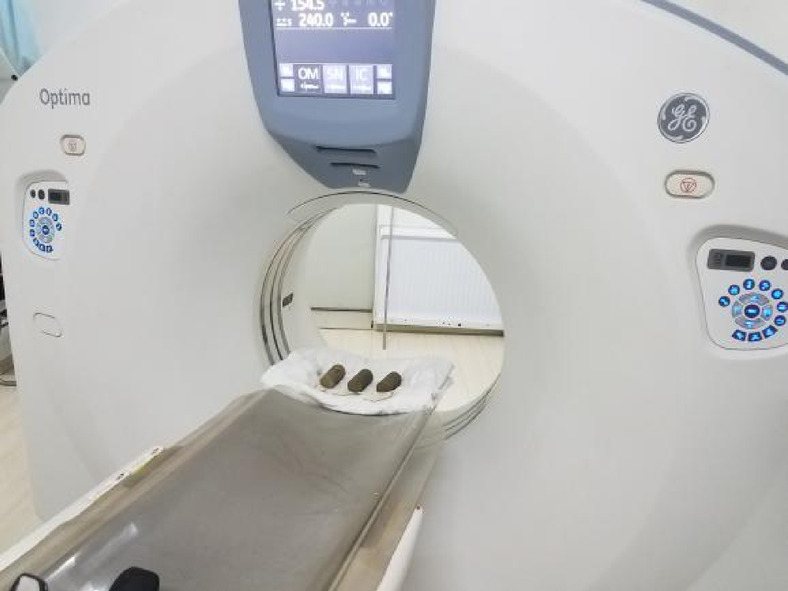
The process of CT scan.

After subjecting the CCSSS to freeze-thaw cycles, CT scanning equipment was utilized to identify its meso-structure under varying freeze-thaw conditions and analyze the internal structural changes. To investigate the impact of freeze-thaw cycles on the mechanical properties of the stabilized soil, a triaxial consolidation undrained (CU) experiment was performed post-CT scan. The experiments applied circumferential pressures of 30, 50, 70, and 100 kPa, reflecting conditions relevant to subgrade structures, with a loading rate of 0.08 mm/min. Figure 5 shows the process of a CT scan.

## Results and discussion

### Mechanical characteristics

**Fig. 6:**
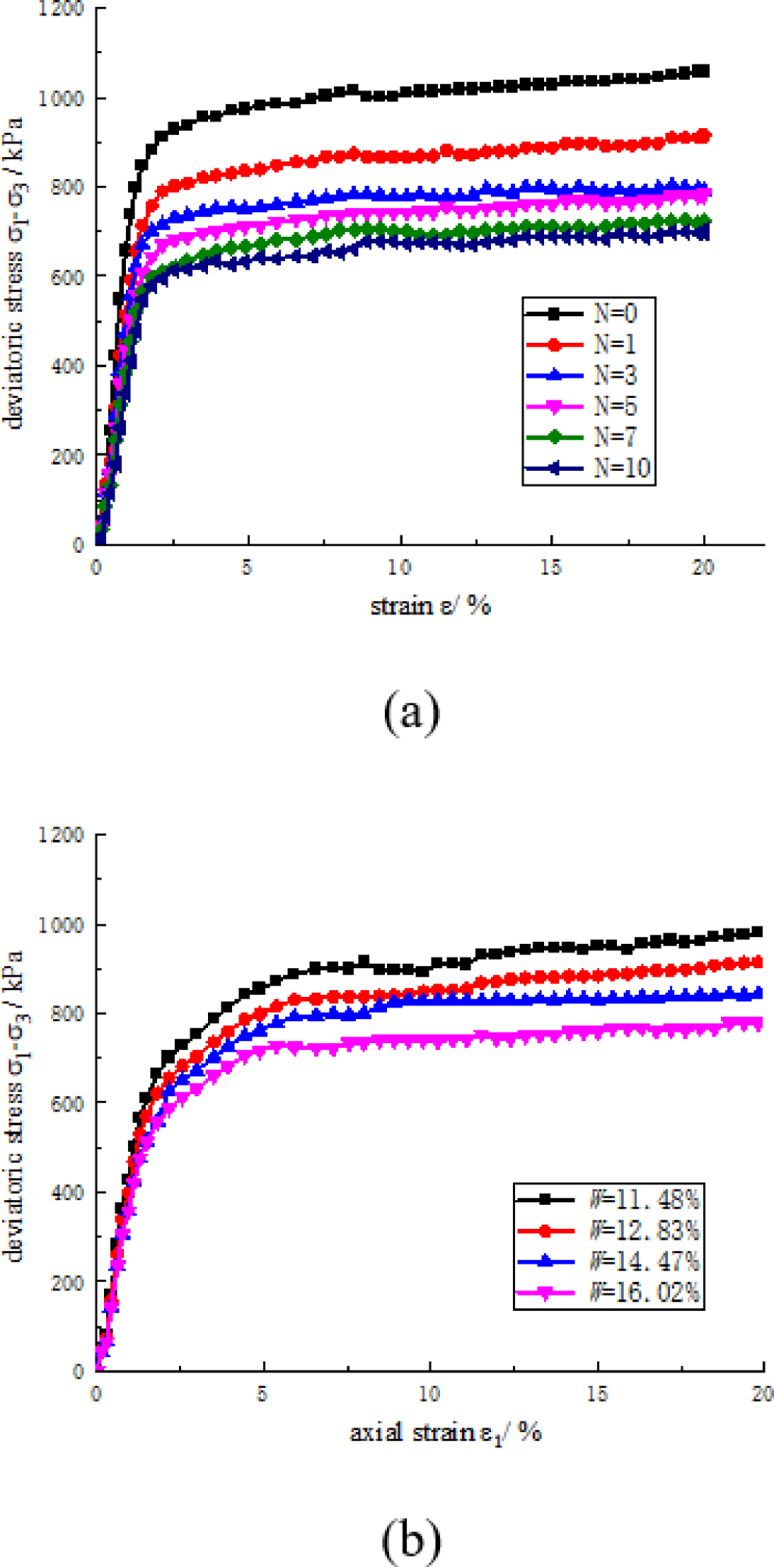
The stress-strain relationship of CCSSS subjected to freeze-thaw cycles. (**a**) Effect of freeze-thaw cycles, (**b**) The effect of water content.

Figure 6 illustrates the stress-strain relationship of CCSSS subjected to varying freeze-thaw conditions. In Fig. 6a, the stress-strain curve of the CCSSS diminished progressively with increasing freeze-thaw cycles, particularly highlighting the pronounced impact of the initial 5 cycles on the CCSSS. This reduction is attributed to the volume expansion resulting from the repeated freezing of pore water within the soil. Consequently, a frost heave force is generated, leading to the compression and deformation of soil particles. This process expands and creates new pores, ultimately disrupting the cemented soil skeleton of CCSSS. Concurrently, thawing facilitates water in sandstone pores redistribution, moving from larger to smaller pores, causing more damaged zones from the frost heave^40^. Figure 6b demonstrates a gradual decrease in the stress-strain curve of the CCSSS with rising water content, exhibiting a slightly accelerated trend of change. The decrease is attributed to elevated water content facilitating more pore water participating in frost heave and thaw damage, thereby amplifying the structural damage efficiency on the soil skeleton of freeze-thaw cycles.

During various freeze-thaw conditions, the deviatoric stress of CCSSS incrementally increased with the increase of axial strain until reaching a stabilized level. The stress-strain relationship of CCSSS samples exhibited strain-hardening properties, with the stress at 15% strain considered as the shear strength of the stabilized soil.

Strain energy density represents the strain energy stored per unit volume of solid material, while failure strain energy density specifically denotes the strain energy density immediately preceding material failure. Its physical significance lies in characterizing the energy absorption capacity of a solid at the point of structural failure.

According to the definition, failure strain energy density can be represented as:

1 $$\upsilon = \int_{0}^{{\varepsilon _{r} }} {\frac{{\sigma _{1} - \sigma _{3} }}{{1 - \varepsilon }}} d\varepsilon$$

In the equation, *υ* represents failure strain energy density, *σ*_1_ represents axial stress, *σ*_3_ represents confining pressure, *ε* represents strain, and *ε*_*r*_ represents failure strain, this paper designed that failure occurs when the strain reaches 15%.

**Fig. 7:**
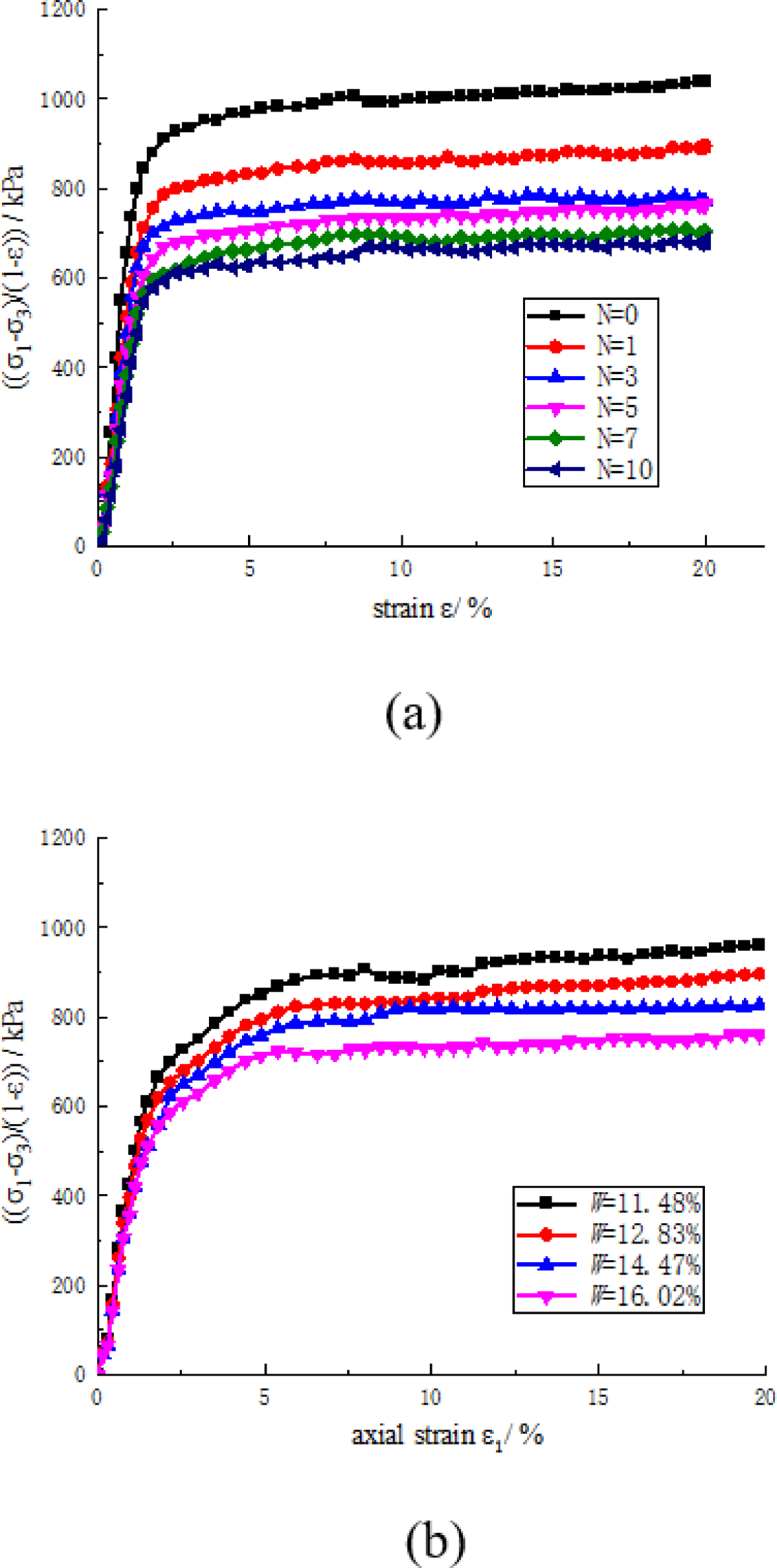
Stress-strain curve after processing stress vertical ordinate. (**a**) Effect of freeze-thaw cycles, (**b**) The effect of water content.

According to Eq. (1), when the stress-strain curve is processed into the form shown in Fig. 7, the integral of this curve along the horizontal axis from 0 to the failure strain corresponds to the failure strain energy density.

Figure 7 demonstrates that the failure strain energy density decreased with increasing freeze-thaw cycles and water content. The samples exhibited a strain energy density of 6366.29 kPa before freeze-thaw cycling, which decreased to 4034.74 kPa after 10 cycles, representing a decrease of 36.62%. Notably, the failure strain energy density reduction caused by the initial five cycles accounted for 75.87% of the total observed decrease. The decrease is attributed to freeze-thaw cycles causing damage to the cemented soil skeleton, thereby the samples stored diminishing energy before the destruction. The group of water content 11.48% showed strain energy density of 5171.7 kPa while diminishing to 4368.7 kPa at the group of water content 16.02%, representing a decrease of 15.53%. The diminishing is attributed to while water content increases, the damage to the cemented soil skeleton caused by pore water is enhanced, thereby the ability of soil to store strain energy diminishes rapidly.

**Fig. 8:**
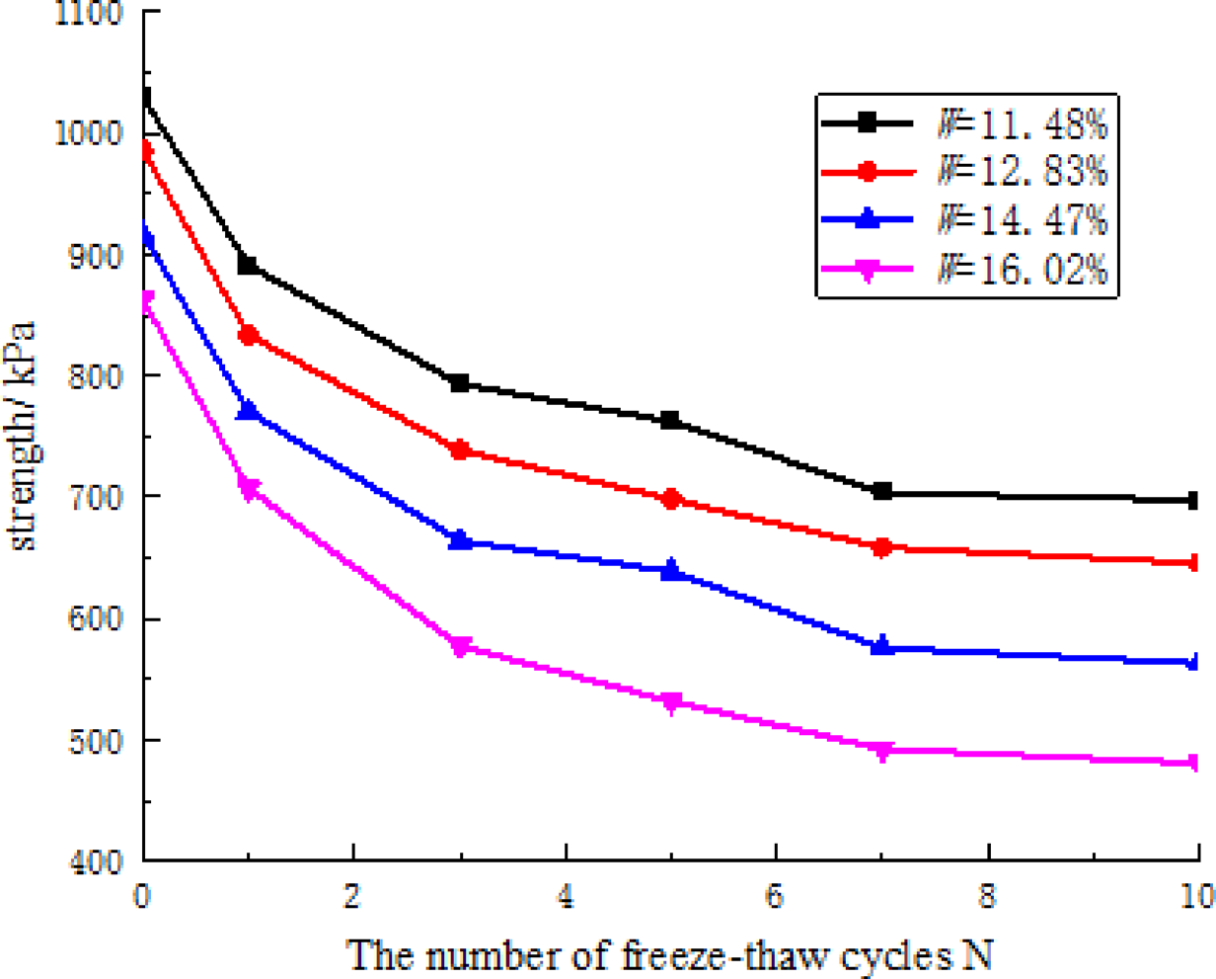
Correlation between freeze-thaw cycles and the shear strength of CCSSS.

Figure 8 illustrates the correlation between the number of freeze-thaw cycles and the shear strength of CCSSS, showing that with an increase in the number of freeze-thaw cycles, the shear strength of CCSSS gradually decreased in an approximately exponential manner. The initial 5 freeze-thaw cycles exhibited more pronounced effects, resulting in a maximum strength loss of 330.75 kPa at a water content of 16.02%, representing 86.6% of the total loss. This damage occurs because as the number of freeze-thaw cycles increases, the pore distribution of CCSSS deteriorates, causing cumulative damage to the microstructure, which ultimately leads to a reduction in strength. Furthermore, the magnitude of strength loss in CCSSS induced by freeze-thaw cycles intensified with increasing water content. Specifically, at a water content of 16.02% (saturation), the strength loss of the stabilized soil after 10 freeze-thaw cycles was 381.88 kPa, indicating a growth of 49.9 kPa compared to samples with a water content of 11.48%. This is because the higher the water content, the greater the destructive impact of freeze-thaw cycles on the soil microstructure, leading to a more rapid decline in soil strength.

**Fig. 9:**
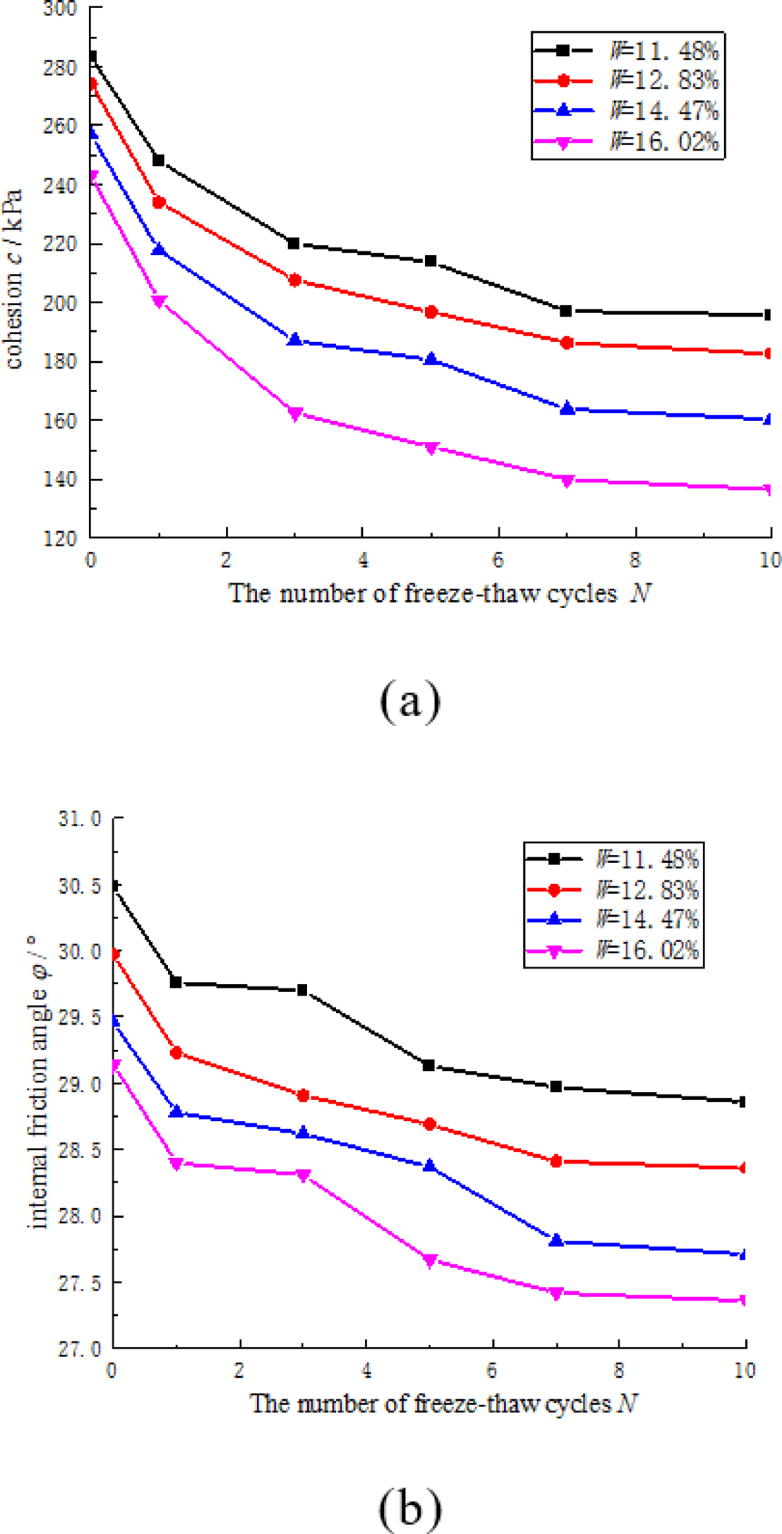
Correlation between freeze-thaw cycles and the shear strength parameters of CCSSS. (**a**) cohesion, (**b**) internal friction angle.

Figure 9 illustrates the correlation between the shear strength parameters of CCSSS and the number of freeze-thaw cycles. The freeze-thaw cycles significantly impacted the shear strength parameters of the CCSSS sample. Specifically, the cohesion and internal friction angle of the CCSSS gradually decreased with an increasing number of freeze-thaw cycles. Notably, the initial 5 freeze-thaw cycles exhibited a more pronounced effect, while the impact stabilized after 7 cycles. The freeze-thaw cycles notably affected the cohesion of CCSSS, with the maximum cohesion decrease of 107.13 kPa observed after 10 freeze-thaw cycles when the initial water content reached 16.02% (saturation), representing a decrease of 45.6%. The cohesion of soil primarily arises from the friction and cementation between particles^21^. As the number of freeze-thaw cycles increases, the enlargement and multiplication of pores diminish the cementation and friction between particles, and disrupt the cementation framework, thereby decreasing the cohesion. In contrast, the impact on the internal friction angle is limited, with a maximum value of 2.15° showing a decrease of 7.3%.

The escalation of initial water content intensifies the influence of freeze-thaw cycles on the shear strength parameters of CCSSS. With the increase in the sample’s initial water content, the loss magnitude of both cohesion and internal friction angle of the CCSSS gradually rose with slight acceleration trends. At an initial water content of 11.48%, the sample undergoes a cohesion decay of 82.53 kPa and a 1.44° decrease in the internal friction angle after 10 freeze-thaw cycles. Upon reaching a water content of 16.02% (saturation), the sample shows a reduction in cohesion of 107.13 kPa and a 2.15° decrease in the internal friction angle following 10 freeze-thaw cycles. As the water content reaches saturation, the reduction in cohesion increases by 18.85 kPa, while the decline in the internal friction angle rises by 0.71°. The reduction of cohesion is due to the fact that as the water content increases, more pore water participates in frost heave, leading to an increase in pores. Thereby, the cementation and friction were diminished between particles, resulting in a greater decrease in cohesion.

### Meso-structure

The X-ray absorption of substances with varying densities exhibits significant variation, therefore the density alterations during the freeze-thaw process are described by the CT value. The correlation between the density of the CCSSS and the CT value before and after freeze-thaw cycles can be represented as:

2 $$\rho = \frac{{\rho _{0} (1000 + H)}}{{1000 + H_{0} }}$$

In the equation, *ρ*_*0*_ and *ρ* represent the density of the soil sample before and after freeze-thaw cycles, *H*_*0*_ and *H* represent the CT values of the soil sample before and after freeze-thaw cycles.

**Fig. 10:**
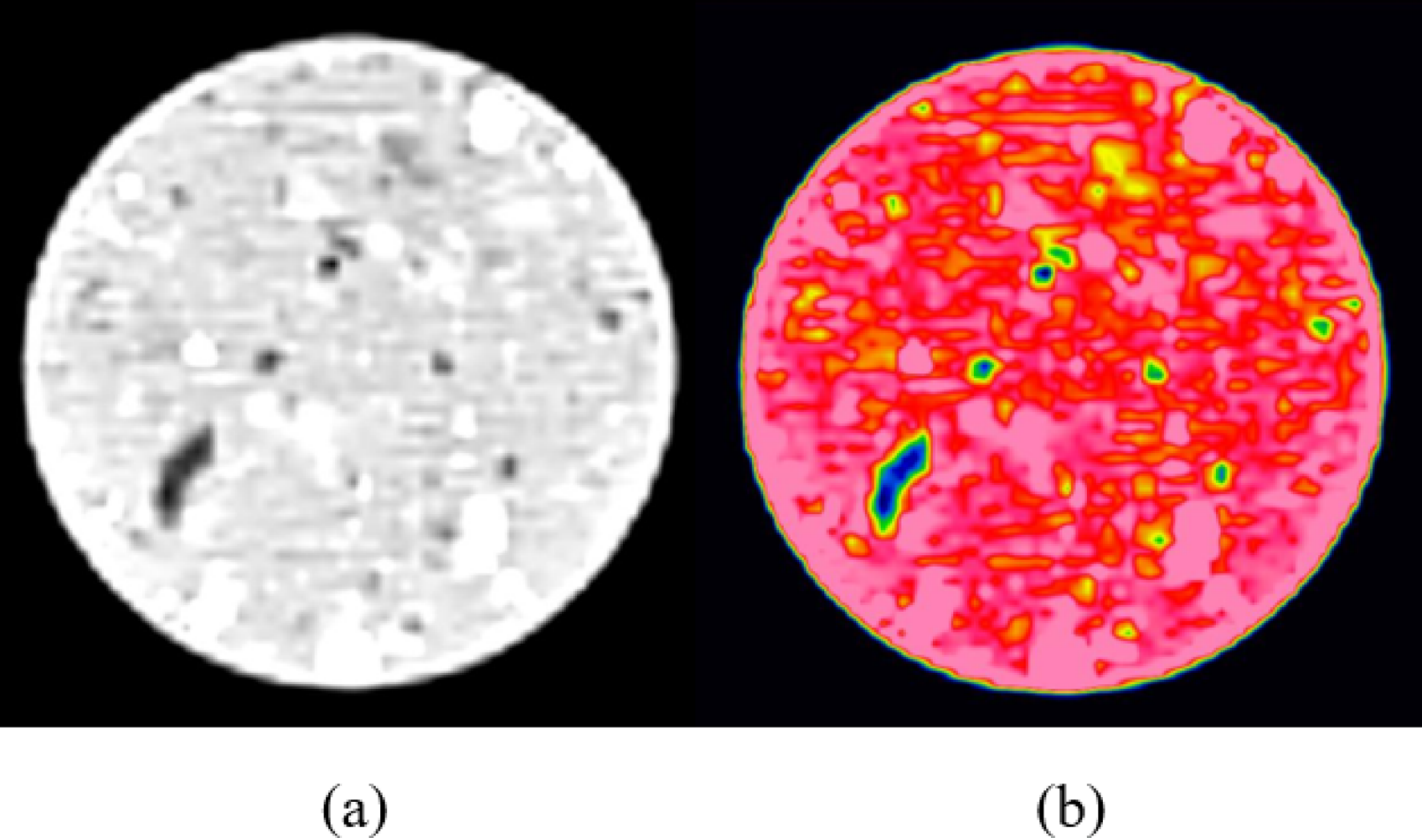
Pseudocoloring of CT Scan Images. (**a**) before pseudocoloring, (**b**) after pseudocoloring.

CT scans produce grayscale images, which were enhanced for improved region differentiation through pseudocoloring. The images before and after pseudocoloring are shown in the (Fig. 10).

**Fig. 11:**
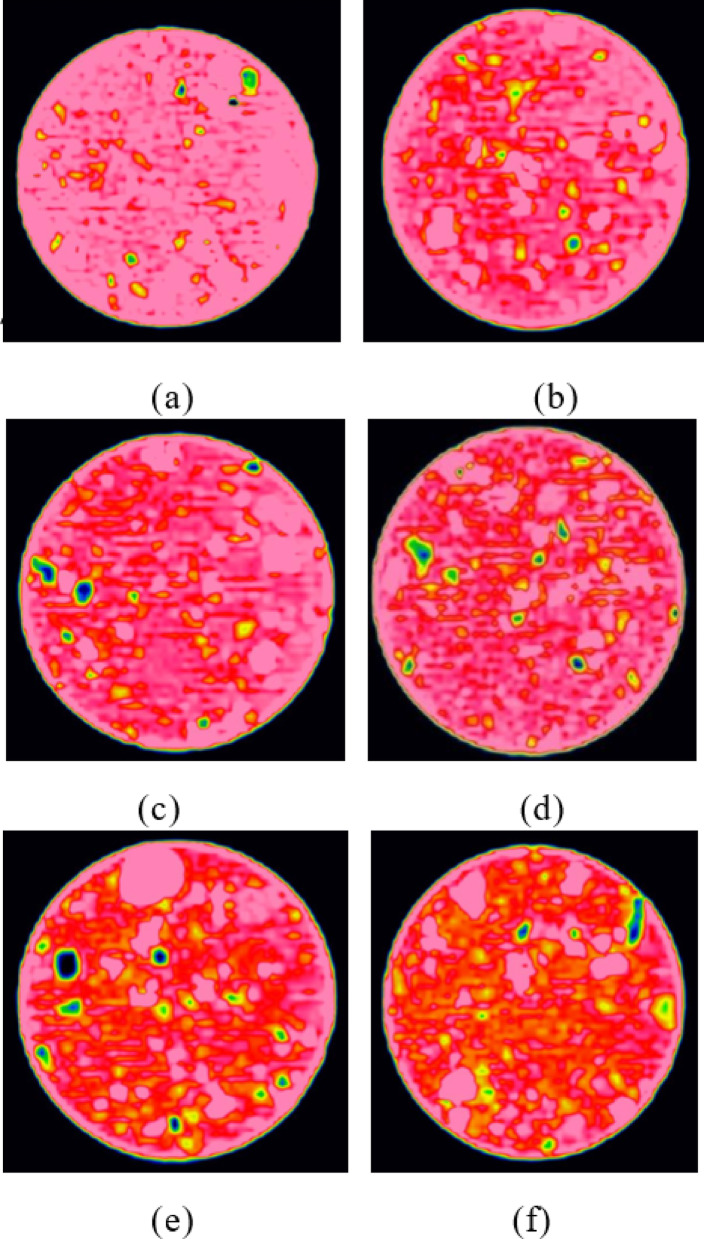
The impact of freeze-thaw cycles on the development of pore structure in sample. (**a**) 0 freeze-thaw cycles, (**b**) 1 freeze-thaw cycles, (**c**) 3 freeze-thaw cycles, (**d**) 5 freeze-thaw cycles, (**e**) 7 freeze-thaw cycles, (**f**) 10 freeze-thaw cycles.

The CT scan images were processed using MATLAB to analyze the distribution pattern of CT values within the images. The images were divided into 4 regions based on CT value distribution: high-density region (2500 ~ 1400 Hu), medium-density region (1400 ~ 700 Hu), low-density region (0 ~ 700 Hu), and pore region (0~-700 Hu), with the low-density and pore regions collectively referred to as the damaged area. Figure 11 illustrates the evolution of the meso-structure of CCSSS under different freeze-thaw cycles.

Figure 11 demonstrates that freeze-thaw action exacerbated the development of pores and cracks within CCSSS. As the number of freeze-thaw cycles increases, the damaged area of the CCSSS gradually expands. The impact of freeze-thaw cycles on the internal structure of the CCSSS is primarily concentrated within the initial 5 cycles, resulting in a 16.28% increase in the damaged area, later the impact diminished and stabilized. This phenomenon is attributed to the formation of numerous cracks due to water freezing inside the CCSSS samples. With an increase in the number of freeze-thaw cycles, these cracks continue to develop, causing significant damage to the internal structure of the stabilized soil. However, the enclosed environment restricted the water supply, leading to a gradual stabilization of this destructive process.

**Fig. 12:**
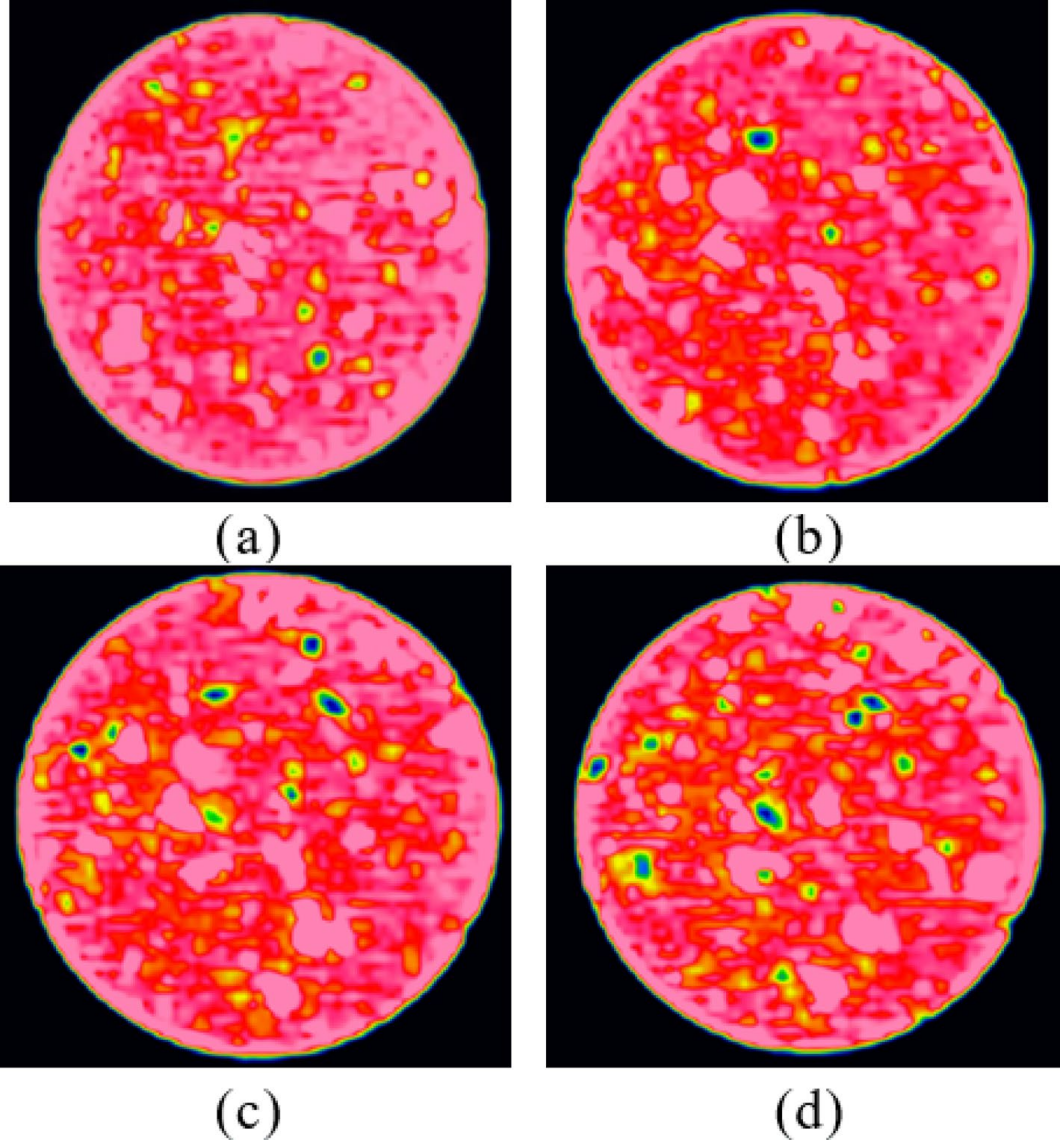
The impact of water content on the development of pore structure in sample. (**a**) *W* = 11.48%, (**b**) *W* = 12.83%, (**c**) *W* = 14.47%, (**d**) *W* = 16.02%.

Figure 12 illustrates the impact of water content on the internal structure of CCSSS sample. A marked positive correlation was observed that with elevated water content, the damaged area of the soil gradually increased, showing a slight accelerating trend. When the initial water content of the samples reached 16.02% (saturation), the damaged area increased by 6.06%. This indicates that an increase in water content amplified the damage to the internal structure of CCSSS caused by freeze-thaw cycles. While the stabilized soil reaches saturation, the internal pores are filled with free water, leading to volume expansion upon freezing, resulting in the enlargement and accelerated development of pores.

### The fractal characteristics

The fractal theory is one of the primary methods for describing the self-similarity and complexity of images, and it has been proven to be applicable in studying the pore structure of soil^41–44^. The fractal dimension characterizes the complexity and irregularity of pores within the soil, and it can be calculated using methods such as the box-counting dimension method, grayscale interpolation method, and double blanket method. Considering the granular nature of CCSSS, this study employed the box-counting dimension method to determine the fractal dimensions of various regions. The calculation method is as follows:

3 $$D = \mathop {\lim }\limits_{{r \to \infty }} \frac{{\ln \;N_{r} }}{{\ln \,\;r}}$$

In the equation, *r* represents the side length of the square box, *Nr* represents the total number of boxes.

The impact of freeze-thaw cycles on the meso-structure of stabilized soil is significant, with varying laws of change in the fractal dimensions of different regions. By considering both the fractal dimensions and the area of each region, the fractal dimension of the damaged area can be represented using Eq. (4).

4 $$D_{S} = \frac{{D_{L} \cdot A_{L} + D_{P} \cdot A_{P} }}{{A_{L} + A_{P} }}$$

In the equation, *D*_*L*_ represents the fractal dimension of the low-density region of the soil sample, *A*_*L*_ denotes the area of the low-density region, *D*_*P*_ signifies the fractal dimension of the pore region of the soil sample, and *A*_*P*_ indicates the area of the pore region.

**Fig. 13:**
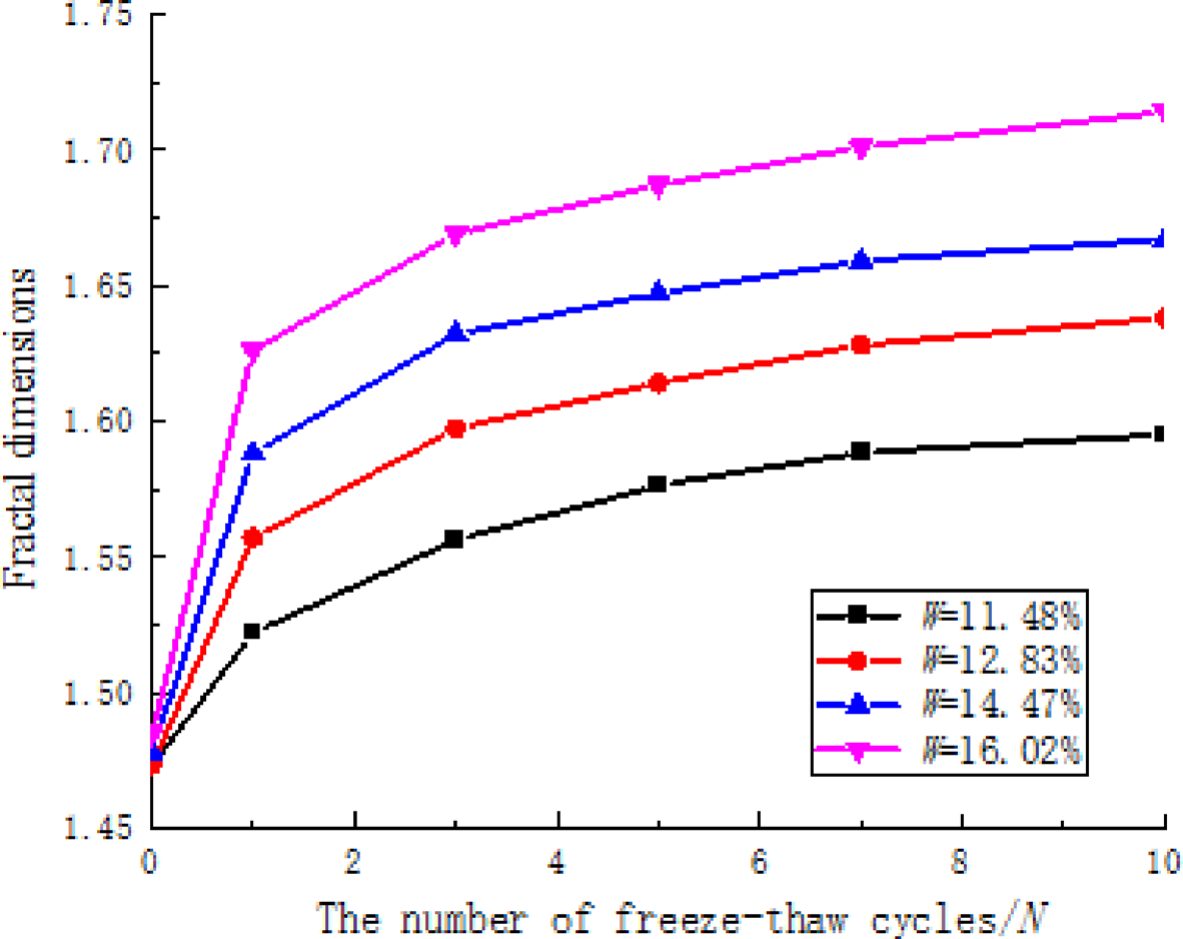
Correlation between freeze-thaw cycles and fractal dimensions of damaged area.

The CT scan images were imported into MATLAB software for processing. Based on the variations in CT values within the images, the morphological characteristics of the low-density and pore regions were extracted separately, and the fractal dimensions of the low-density and pore regions of CCSSS were calculated. By utilizing Eq. (4), the relationship between the fractal dimension of the damaged area of the stabilized soil and the number of freeze-thaw cycles was determined for different numbers of freeze-thaw cycles, as illustrated in (Fig. 13).

Figure 13 illustrates that under different freeze-thaw conditions, the fractal dimension range of the damaged area of CCSSS is between 1.472 and 1.714. With an increase in the number of freeze-thaw cycles, the fractal dimension of the damaged area of the stabilized soil gradually increased. The impact of the first 5 freeze-thaw cycles was particularly pronounced, but this effect diminished after 7 cycles, leading to a more gradual transition in the region. This indicates that freeze-thaw cycles promote the continuous development of pores and cracks in the stabilized soil samples, increasing their complexity, spatial disorder, self-similarity, and roughness of pores.

### Analysis of damage evolution

**Fig. 14:**
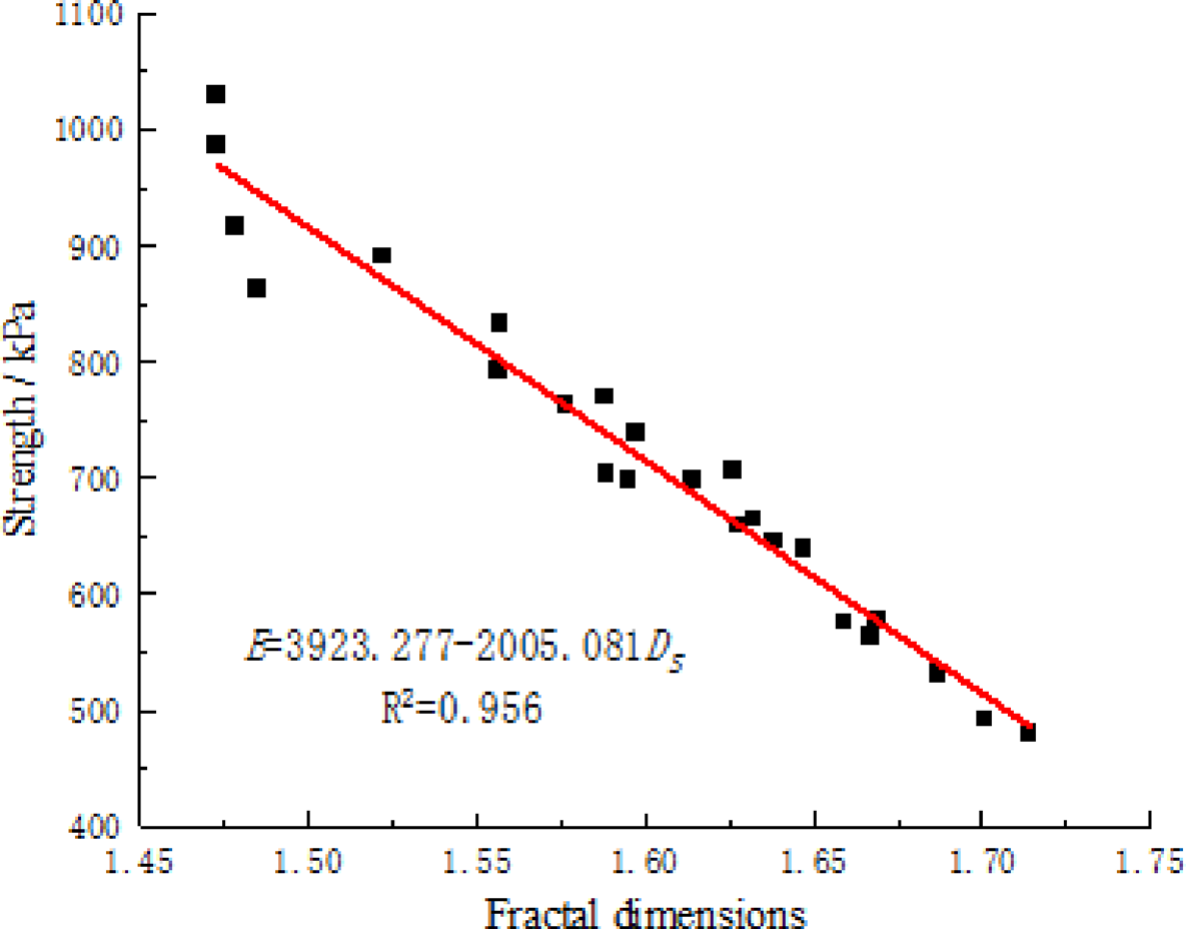
Correlation between strength of CCSSS and fractal dimensions of damaged area.

Figure 14 illustrates the relationship between the shear strength and fractal dimension of the damaged area of CCSSS under different freeze-thaw cycle experiment conditions. It is evident that as the fractal dimension increased, the shear strength of the stabilized soil decreased gradually, showing a clear linear correlation between the two. This indicates a certain connection between the evolution of the meso-structure of CCSSS and its macroscopic physical parameters, with the intensification of freeze-thaw action, the internal damaged area of the stabilized soil gradually expands and an accumulation of damage increases leading to a noticeable characteristic strength deterioration.

Damage refers to the extent of mechanical behavior deterioration of materials under the influence of loads and external environmental conditions. The damage variable of stabilized soil under freeze-thaw action can be expressed as:

5 $$S = 1 - \frac{{f_{N} }}{{f_{0} }}$$

In the equation, *f*_0_ represents the initial strength of the stabilized soil(kPa), *f*_N_ denotes the strength of the stabilized soil after undergoing freeze-thaw cycles(kPa).

**Fig. 15:**
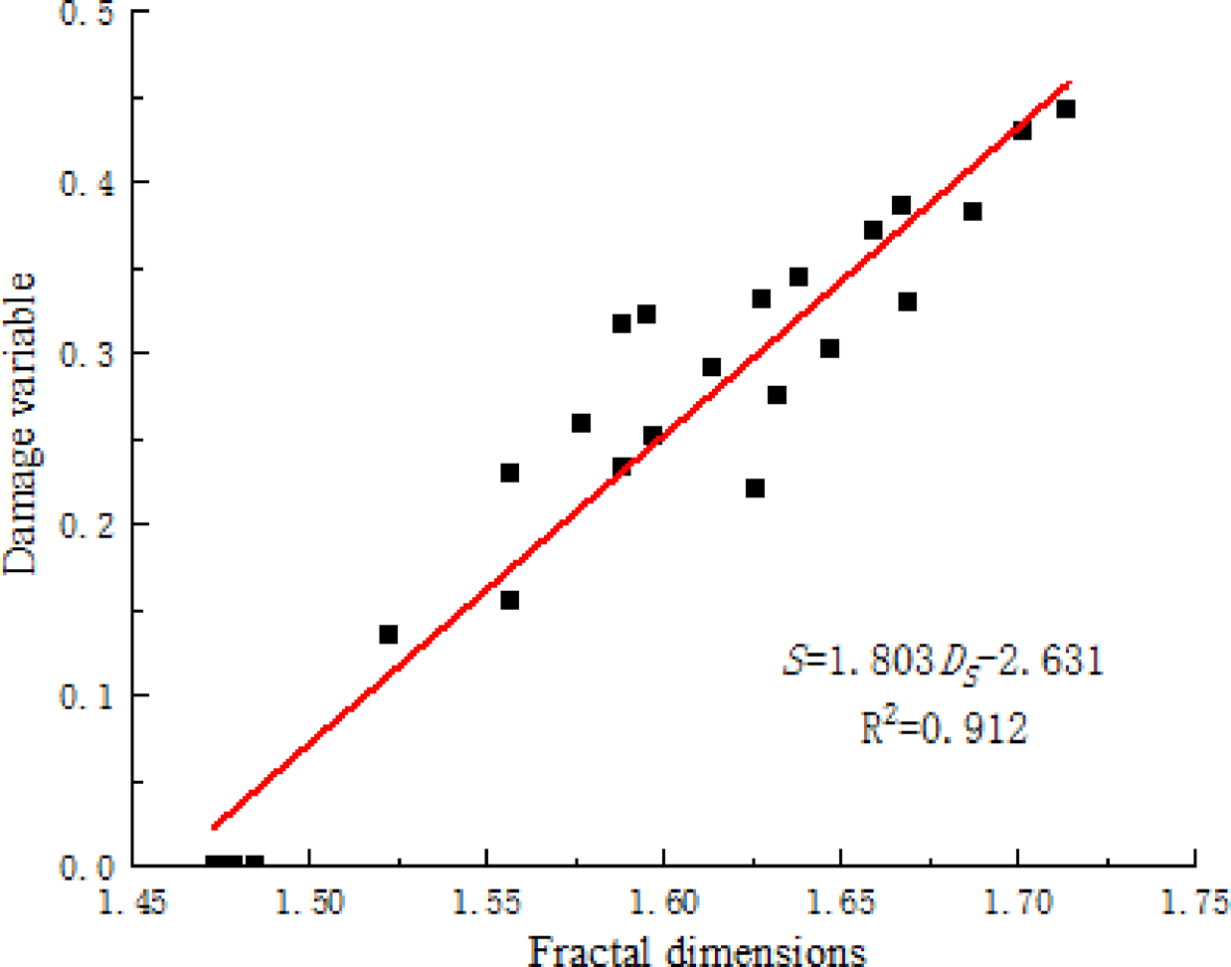
Correlation between damage variable and fractal dimension of the damaged area.

Figure 15 illustrates the relationship between the damage variable and the fractal dimension of the damaged area of the stabilized soil sample under different freeze-thaw cycle experiment conditions. As the fractal dimension of the damaged area increased, the extent of damage in the stabilized soil specimens gradually intensified, showing a roughly linear correlation between the two. Combining the results of CT scanning experiments reveals that freeze-thaw cycles have a significant impact on the internal structure of the stabilized soil, leading to the continuous development of cracks and expansion of the damaged area. The complexity and roughness of the damaged area morphology increase, thereby exacerbating the degree of damage.

## Conclusion

This study investigates the shear strength, meso-structure evolution, and fractal characteristics of CCSSS under freeze-thaw conditions through triaxial compression experiments and CT scanning experiments. The main conclusions drawn are as follows:

1. The shear strength of CCSSS shows exponential decay with an increase in the number of freeze-thaw cycles. The influence of the first 5 cycles was particularly pronounced, resulting in a maximum strength loss of 330.75 kPa at a water content of 16.02%, accounting for 86.6% of the total loss. As the water content increased, the impact of freeze-thaw cycles on the strength of CCSSS intensified. Freeze-thaw cycles significantly affected the strain energy density and cohesion of CCSSS. A maximum cohesion reduction of 107.13 kPa was observed after 10 cycles, representing a decrease of 45.6%, with strain energy density decreasing by 36.62%. The effect on the internal friction angle is not prominent.
2. The freeze-thaw action intensifies the development of internal pores and cracks in CCSSS. With an increase in the number of freeze-thaw cycles, the damaged area of the stabilized soil gradually enlarged. The impact of the first 5 freeze-thaw cycles was particularly notable, leading to a maximum increase of 16.28% in the damaged area. Moreover, as the water content rose, the damaged area of CCSSS exhibited an accelerating growth trend, when the initial water content of the specimen reached 16.02% (saturation), the damaged area expanded by 6.06%.
3. The fractal dimension is a reliable indicator of the impact of freeze-thaw cycles on the meso-structure of stabilized soil specimens. With the increasing influence of freeze-thaw cycles, the fractal dimension of the damaged area in the stabilized soil gradually rose. There existed a strong linear negative correlation between the fractal dimension of the damaged area and the shear strength, effectively describing the damage induced by freeze-thaw actions and its evolution rule in the soil.

The systematic analysis of the findings in this paper delves into the damage patterns caused by freeze-thaw cycles on subgrade fill, providing a valuable reference for the construction and maintenance of subgrade engineering in seasonal freezing regions. Coal slag is a type of industrial waste, its usage in subgrade fill is beneficial to mitigate environmental pollution and yield economic benefits.

## Data Availability

Data will be made available on request by contacting Mengqi Shi. E-mail: a3702112@163.com.

## Software

Name: OriginPro. Version number: 2022. URL link: https://www.originlab.com/ReleaseNotes/index.aspx?v=2022.
